# Response of *Alternaria* and *Fusarium* Species to Low Precipitation in a Drought-Tolerant Plant in Morocco

**DOI:** 10.1007/s00248-024-02439-3

**Published:** 2024-10-11

**Authors:** Jean Legeay, Sulaimon Basiru, Abdelhadi Ziami, Khaoula Errafii, Mohamed Hijri

**Affiliations:** 1 African Genome Center, University Mohammed VI Polytechnic (UM6P), Ben Guerir, Morocco; 2 Institut de Recherche en Biologie Végétale, Département de Sciences Biologiques, Université de Montréal, Montreal, Quebec Canada

**Keywords:** Fungi, Mycobiome-plant interaction, *Malva sylvsestris*, Dark septate endophytes, Rhizosphere

## Abstract

The plant mycobiome plays a crucial role in the host life cycle, influencing both healthy and diseased states, and is essential for plant tolerance to drought. In this study, we used ITS metabarcoding to investigate the fungal community of the drought-resistant plant *Malva sylvestris* L. in Morocco along a gradient of precipitation, encompassing subhumid and semi-arid environments. We sampled three biotopes: rhizosphere, bulk soil, and root endosphere. Our findings revealed an absence of beta-diversity differences between bulk soil and rhizosphere, indicating that the plant does not selectively influence its rhizosphere mycobiome. Additionally, ASVs belonging to the genus *Alternaria* represented up to 30% of reads in the plant’s roots and correlated with drought (*p* = 0.006), indicating a potential role for this fungal genus in mitigating drought, possibly as part of the dark septate endophyte group. Root staining and microscopic observation revealed extensive colonization by fungal hyphae and microsclerotia-like structures. Furthermore, ASVs identified as *Fusarium equiseti* were also correlated with low precipitation and recognized as a hub taxon in the roots. However, it remains uncertain whether this species is pathogenic or beneficial to the plant. These insights contribute to our understanding of the plant mycobiome’s role in drought tolerance and highlight the importance of specific fungal taxa in supporting plant health under varying environmental conditions. Future research should focus on characterizing these taxa’s functional roles and their interactions with the host plant to further elucidate their contributions to drought resistance.

**Supplementary Information:**

The online version contains supplementary material available at 10.1007/s00248-024-02439-3.

## Introduction

Developing adaptive strategies to address climate change events is unequivocally one of the most pressing challenges of the twenty-first century and beyond. Among these climate-related issues, increasing aridity, prolonged periods of drought, and excessive and erratic rainfall are of major concern [1]. Recent revelations indicate that prolonged drought and erratic rainfall are negatively impacting global food security [2]. In Morocco, long-term drought and erratic rainfall decreased agricultural productivity by approximately 17% in 2022 [3].

Plant mycobiomes—the fungal communities inhabiting plant roots, rhizospheres, and phyllospheres—are an integral component of plant biology that can mediate plant adaptation to extreme conditions such as heat and drought. The role of symbiotic fungal associations in plants’ tolerance to abiotic stress has been the focus of many recent studies [4, 5]. Beneficial associations involving mycorrhizal and endophytic fungal communities can enhance the phenotypic and metabolic plasticity of host plants [6, 7], resulting in improved tolerance to stresses such as drought and salinity [8].

A group of endophytic plant root mycobiome known as dark septate endophytes (DSE) have been found to extensively colonize many plant roots including trees and herbaceous plants [9]. Despite the growing awareness about the importance of this fungal group in plant growth promotion, phosphorus acquisition, and drought tolerance in arid ecosystems [10, 11], little is known about their selection, recruitment, and turnover along arid bioclimatic gradients. Although DSE can be identified by the presence of melanized and hyaline septate hyphae, as well as microsclerotia in the intraradical roots [7], high-throughput sequencing techniques are essential to study their diversity and distribution and to identify their role in plant mycobiome assembly and selection.

Environmental perturbations, such as increased warming and drought, can result in a significant turnover of plant root mycobiomes. However, the selective recruitment of specific taxa by host plants in the roots and rhizosphere can complicate the diversity and assembly of fungal communities along specific climatic or ecological gradients. For example, alpha-diversity of the fungal community in the roots and rhizosphere of castor bean (*Ricinus communis*) increased under drought compared to wet conditions; however, the higher selectivity for certain groups in drought conditions led to a greater uniformity of fungal communities, thus reducing species turnover [12]. In another study, Gargouri et al. [13] reported that while *Opuntia ficus-indica* mycobiome exhibited similar alpha-diversity across an aridity gradient, there was a significant difference in the beta-diversity. The relative abundances of major phyla differed between the rhizosphere and endosphere, and distinct hub taxa were recruited at each biotope within different bioclimatic zones [13]. Therefore, further assessment of plant mycobiomes is essential to understand the ecology of plant-associated fungal communities across spatially diverse aridity zones.

*Malva sylvestris* L., commonly known as common mallow, belongs to the Malvaceae family and is widely distributed across the globe. The plant thrives along a broad gradient of aridity and demonstrates resilience to both drought and excessive rainfall [14]. *M. sylvestris* can grow in marginal or resource-poor ecosystem; this property enables the plant to play a role as a pioneering plant in the ecological succession of barren or disturbed habitats. Due to its ability to grow in both wet and dry climates, *M. sylvestris* is also a promising candidate for studying the ecological succession of plant mycobiomes across aridity gradients [15]; however, there is limited information on the ecology of *M. sylvestris* mycobiome. In a recent study conducted by Legeay et al. (2024) [15], *Rhizobium* dominated the root bacteriome of *M. sylvestris*, but this observation was not related to any specific variable in the study. Interestingly, the study also found no significant differences in beta-diversity between the rhizosphere and bulk soil, suggesting that *M. sylvestris* might exhibit weak selective recruitment of bacteriome in its rhizosphere.

Therefore, this study aims to investigate the mycobiome of the plant *M. sylvestris* in both agricultural and natural environments to understand how fungal community structures change, particularly in arid regions, and to identify fungal taxa associated with the plant’s drought tolerance. Based on previous bacterial studies, we hypothesized that (i) *M. sylvestris* plants do not select specific fungal communities in their rhizosphere, and (ii) *M. sylvestris* plants harbor drought-responsive taxa in their roots. To test these hypotheses, we sampled 101 individuals from 13 sites across Morocco, including five agricultural and eight natural areas along a gradient of precipitation. ITS of rRNA gene metabarcoding was employed to investigate fungal communities in the bulk soil, rhizosphere, and root endosphere of *M. sylvestris*.

## Materials and Methods

### Sampling

We conducted a sample campaign on February 21 and 22, 2022, to collect samples of *M. sylvestris* from five agricultural and eight natural sites across Morocco. Different crops, indicated in Table S1, were cultivated in the agricultural sites. In this specific study, the sites were classified as either sub-humid (annual precipitation > 356 mm) or semi-arid (annual precipitation ≤ 356 mm). The eight sub-humid sites had an average annual rainfall of 492 mm and an average annual high temperature of 25 °C, while the five semi-arid sites had an annual rainfall of 323 mm and a mean annual temperature of 27 °C [16]. The threshold of 356 mm/year was chosen to differentiate between sub-humid and semi-arid sites because it represented the median precipitation amount of all sites. However, due to the unevenness of this classification, precipitation levels were considered a quantitative factor for the rest of the analysis, ranging from 291 to 528 mm/year.

At each site, five to six plants were used to produce five root samples and five rhizosphere samples. Additionally, a composite bulk soil sample was created by pooling five original bulk soil samples taken from five points at the sampling site away from *M. sylvestris* plants. This pooling was done because the study mainly focused on plant-soil interactions and was subject to budgetary constraints.

During collection, the plants were categorized into two possible phenotypes: one creeping at the soil surface and one erect with vertical shoot elevation. The plants were collected by extracting the entire plant and its attached rhizosphere using a hoe and were placed in an icepack-cooled container for the duration of the sampling. To produce root and rhizosphere samples, the rhizosphere was collected by shaking the soil from the roots. The roots were then cut from the rest of the plant, washed with tap water to remove soil particles, washed again with sterile demineralized water, and finally lyophilized. Details and a map of the sampled sites are available in Legeay et al. [15].

### Soil Physico-chemical Analyses

To determine the physico-chemical properties of the soil samples, equal amounts of the single bulk soil sample per site and the five rhizosphere soil samples from the same site were blended to create one composite sample for each site. These samples were sent to the AITTC laboratory at the University Mohammed VI Polytechnic (Benguerir, Morocco) for physico-chemical analysis.

### Root Staining and Microscopy Observation of Fungal Structures

Morphological observation of the fungi colonizing *M. sylvestris* roots was done using the root staining procedure based on the ink-vinegar method [17]. In brief, root samples were trimmed into small fragments measuring 1–2 cm and then treated with 10% KOH in a microwave at full power for 2 min to clear them. After clearing, the roots were rinsed with acidified tap water and subsequently stained with 5% ink in vinegar solution. Following staining, the root fragments were mounted on a slide and examined under an optical microscope (ZEISS Axio Imager 2, Professional Labo, Casablanca, Morocco). Microscopic observations were used to confirm the colonization of roots by DSE.

### DNA Extraction, Amplification, and Sequencing

DNA from soil and root samples was extracted using Qiagen PowerSoil Pro and Qiagen DNeasy Plant Pro kits, following the manufacturer’s instructions (Qiagen, Global Diagnostic Distribution, Temara, Morocco) [15]. A 50 µL volume PCR was conducted, targeting the fungal ITS2 region using the HPLC-purified primers CS1_ITS3_KYO2 (5′-*ACACTGACGACATGGTTCTACAGATGAAGAACGYAGYRAA*-3′) and CS2_ITS4 (5′-*TACGGTAGCAGAGACTTGGTCTTCCTCCGCTTATTGATATGC*-3′) (Alpha DNA, Montreal, Canada). The cycling condition follows an initial denaturation of 94 °C for 3 min, followed by 30 cycles of 94 °C for 30 s, 55 °C for 30 s, and 72 °C for 1 min, with a final elongation step at 72 °C for 7 min.

The method for preparing the library for amplicon sequencing is detailed in Legeay et al. (2024) [15]. Briefly, libraries were prepared using AMPure XP beads (Beckman Coulter, Rabat, Morocco), purified, and resuspended in 10 mM Tris (pH 8.5). Following PCR amplification of ITS, ligation was carried out using unique indexing primers (Fluidigm, Markham, ON, Canada). Indexed amplicons were then purified, quantified with the Qubit DNA HS kit (Thermo-Fisher Scientific, Rabat, Morocco), and pooled. Sequencing was performed using the MiSeq Reagent Kit V3 (600 cycles) (Illumina, MegaFlex, Casablanca, Morocco). The MiSeq run consisted of 2 × 300 bp paired-end sequencing.

### Bioinformatics and Statistical Analysis

The raw reads were analyzed using the DADA2 [18] package of R. Both forward and reverse reads were truncated of 50 pb at their end, because of low quality in this part of the reads. Reads with an expected error rate of more than 3 were discarded. The resulting ASVs were then assigned through BLAST on the fungal taxonomic database UNITE, following the “assignTaxonomy” command.

The alpha-diversity of the communities was computed and analyzed using the *phyloseq* [19] package of R. ANOVA tests were conducted using the “aov” command on the Shannon and Simpson indices for qualitative factors, including agricultural usage of the sites, creeping/erect phenotype of the plants, and the different biotopes (bulk soil, rhizosphere, and root endosphere) as fixed factors. The sites were treated as a random factor. For quantitative factors, such as chemical characteristics and mean annual precipitation, a Pearson correlation test was performed between the Shannon and Simpson indices and the quantitative factors.

The beta-diversity was tested using the *vegan* package [19] with ADONIS PERMANOVA tests, focusing on the same qualitative factors previously described and employing 999 permutations. For quantitative factors, such as chemical characteristics and mean annual precipitation, we performed a constrained correspondence analysis with these factors as constraints, followed by an ANOVA-like permutation test to evaluate the significance of the constraints. Principal coordinates analysis (PCoA) was conducted using Bray–Curtis distances between samples.

When comparing bulk soil samples to root and rhizosphere samples, we randomly selected one sample per site from the root and rhizosphere samples to match the number of bulk soil samples.

Core taxa were identified for each qualitative parameter as the taxa present in more than 90% of the samples of said parameter, regardless of the abundance of the taxa in any individual samples.

Before the analysis of co-occurrence networks and random forest models, the dataset was trimmed to only retain taxa representing more than 0.1% of the sum of all reads. Co-occurrence networks were calculated and graphically represented using the packages *SpiecEasi* [20] and *igraph* [21] of R. Betweenness was calculated for all ASVs of the network using the betweenness function of *igraph*. The hub taxa were designated as the taxa in the top 5% in terms of value of betweenness.

The best predictors ASVs for aridity and agricultural usage were described using random forest models, through the *randomforest* package of R [22] with an iteration of 100 trees.

To try to identify ASVs pertaining to putative dark septate, the entire set of sequences found by a search on NCBI using the keywords (ITS + dark + septate) was downloaded, and ASVs from this study were then aligned using BLAST on this database. ASVs with an *e*-value of less than 10^−25^ were then considered putative dark septates.

## Results

### Soil Chemical Characteristics and Plant Phenotype

Table S1 provides the total amounts of nitrogen (N), carbon (C), phosphorus (P), and potassium (K) for each sample. The K and P contents of the soil were strongly correlated with agricultural soils (*p* = 1.49^−6^ and *p* = 2.54^−6^, respectively). However, the total C and N contents were not significantly correlated with agricultural soils (*p* = 0.84 and *p* = 0.60, respectively). There were no significant differences in chemical contents between rhizosphere and bulk soil (*p* = 0.25). The erected phenotype was more likely to be observed in regions with higher precipitation levels (*p* < 0.001) and higher amounts of total C (*p* = 0.02) compared to the creeping phenotype.

### Raw Sequencing of Fungal ITS

**Fig. 1:**
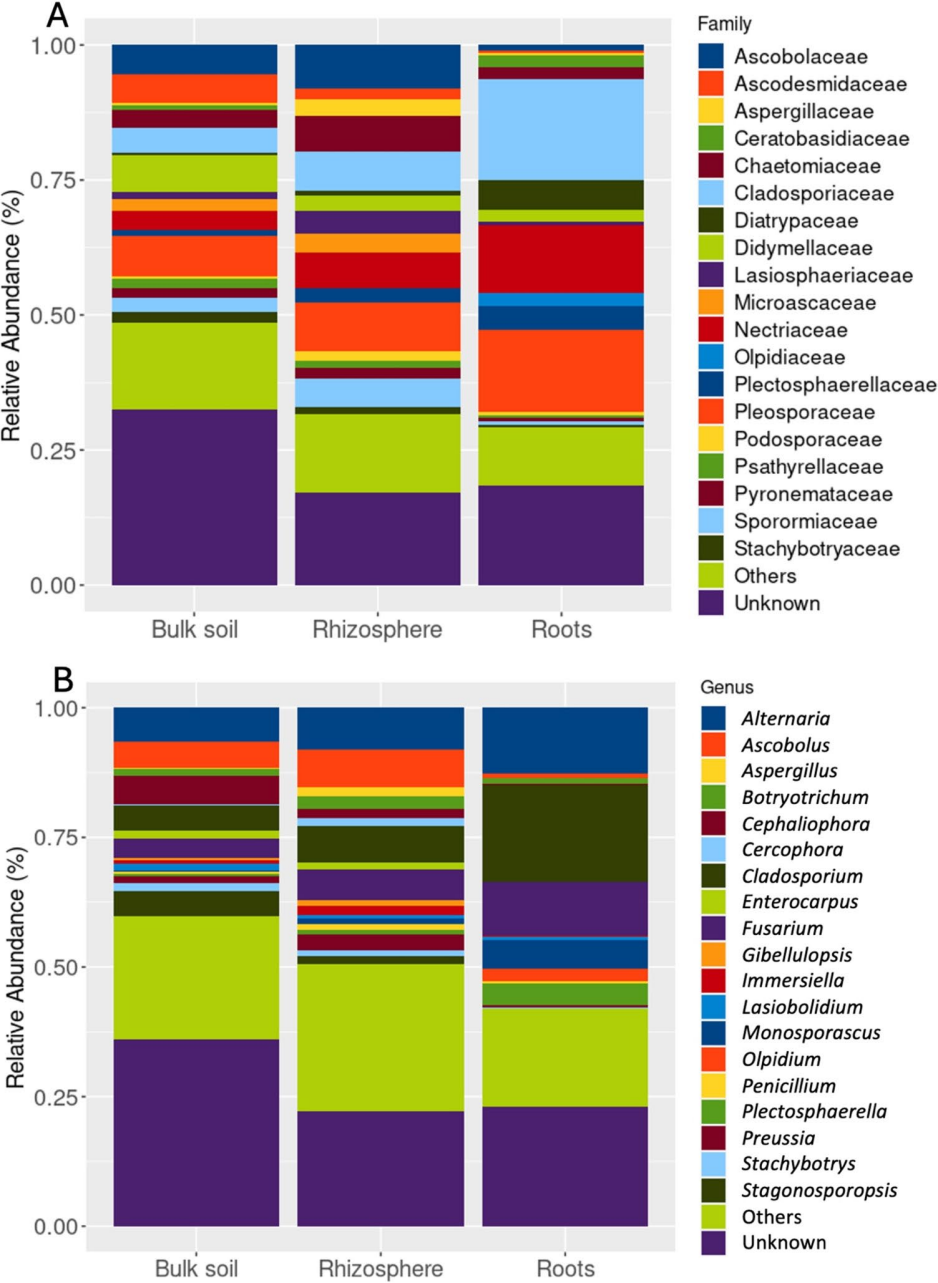
Relative abundances in the three biotopes of families (**A**) and genera (**B**)

A total of 5242 fungal ASVs were identified, spanning 537 species, 390 genera, 196 families, 87 orders, 38 classes, and 15 phyla. The most abundant families were *Cladosporiaceae* (11%) and *Pleosporaceae* (11%), with ASVs from unknown families comprising 18% of the reads (Fig. 1). The most prevalent genera were *Cladosporium* (19%), *Alternaria* (12%), *Fusarium* (10%), *Ascobolus* (7%), and *Cephaliophora* (5%) (Fig. 1). At the genus level, 73 genera were common across all biotopes. At the ASV level, no ASVs were shared between soil samples and roots, while 299 ASVs were shared between bulk soil and the rhizosphere.

### Alpha- and Beta-Diversities

**Fig. 2:**
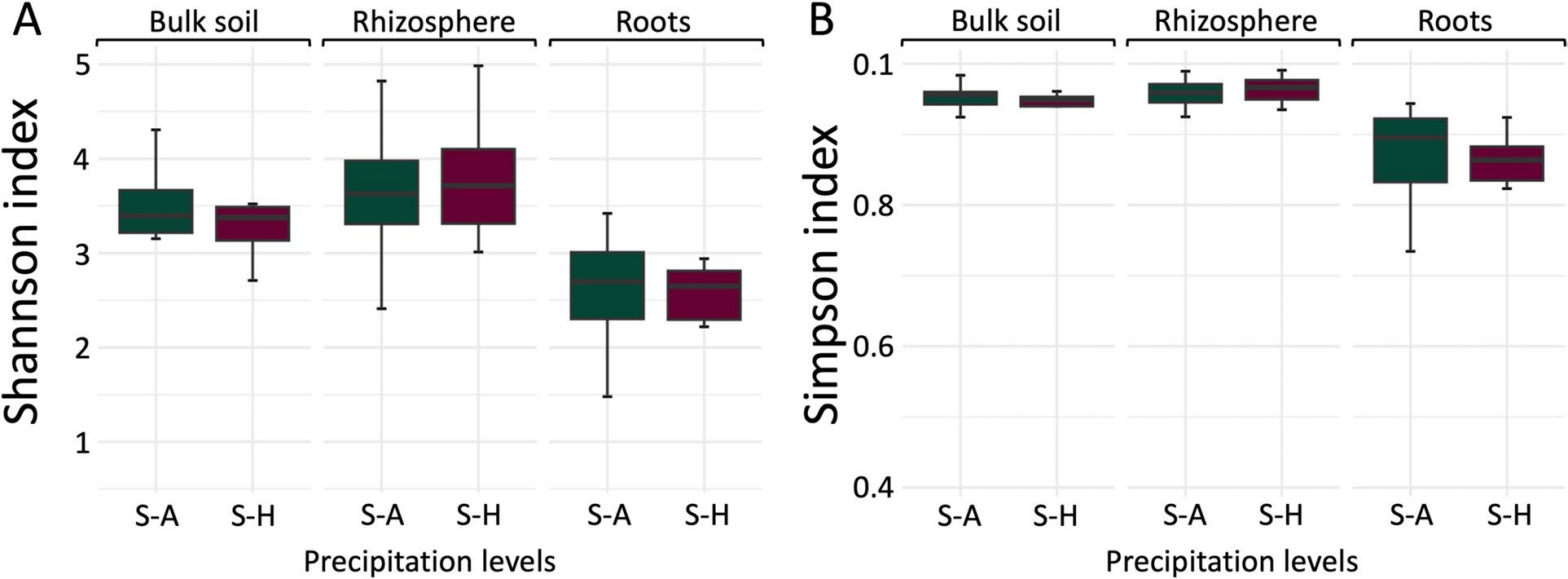
Shannon (**A**) and Simpson (**B**) indices for each biotope (bulk soil, rhizosphere, and roots) across two precipitation levels (semi-arid and subhumid). S-A represents semi-arid environments, while S–H denotes subhumid environments

After subsetting the rhizosphere and root samples to one per site, the alpha-diversity indices in soil samples were significantly higher than those in the roots (*p* < 0.001 for both Shannon and Simpson indices) (Fig. 2). There was no significant difference in alpha-diversity between bulk and rhizosphere soil samples (*p* = 0.1 for Shannon and *p* = 0.08 for Simpson).

In the root endosphere, the Kruskal–Wallis test of Shannon and Simpson diversity indices showed no differences based on agricultural versus natural sites, plant phenotypes, precipitation levels, or total C, N, K, or P (Table 1). However, in soil samples, a negative correlation was found between both Shannon and Simpson indices and total K (*p* = 0.03 and *p* = 0.007, respectively) (Table 2).

**Table 1:** The *p*-values resulting from the Kruskal–Wallis test applied to Shannon and Simpson indices, considering various factors, were analyzed in both soil and root samples. Since there was no significant difference in alpha-diversity between bulk soil and rhizosphere samples, they were combined and treated as soil samples

Factor	Biotope	Shannon	Simpson
Total P	Soil	0.45	0.16
	Roots	0.07	0.05
Total C	Soil	0.71	0.9
	Roots	0.4	0.45
Total K	Soil	0.03*	0.007**
	Roots	0.05	0.09
Total N	Soil	0.93	0.89
	Roots	0.18	0.27
Precipitation	Soil	0.37	0.47
	Roots	0.81	0.92
Agricultural use	Soil	0.79	0.14
	Roots	0.5	0.498
Crop	Soil	0.29	0.43
	Roots	0.15	0.15

Significance levels (*p*-value) are shown and asterisks (*p ≤ 0.05, **p ≤ 0.01) indicate a significant difference in factors between alpha diversity indexes and biotopes

**Table 2:** *p*-values of the effects of various factors on the beta-diversity in the rhizosphere and the roots

Factor	Biotope	*p*-value	*R*^2^ value
Total P	Rhizosphere	0.001***	0.02
	Bulk soil	0.02**	0.02
	Roots	0.03*	0.02
Total C	Rhizosphere	0.001***	0.01
	Bulk soil	0.207	0.01
	Roots	0.05**	0.02
Total K	Rhizosphere	0.001***	0.02
	Bulk soil	0.121	0.01
	Roots	0.03*	0.02
Total N	Rhizosphere	0.001***	0.02
	Bulk soil	0.093	0.01
	Roots	0.024*	0.02
Precipitation	Rhizosphere	0.02**	0.01
	Bulk soil	0.001***	0,08
	Roots	0.272	0.02
Agricultural use	Rhizosphere	0.001***	0.04
	Bulk soil	0.001***	0.11
	Roots	0.124	0.02
Plant phenotype	Rhizosphere	0.019*	0.02
	Roots	0.06	0.04
Precipitation*Agricultural use	Rhizosphere	0.004**	
	Bulk soil	0.362	
	Roots	0.417	
Precipitation*Phenotype	Rhizosphere	0.966	
	Roots	0.027*	
Phenotype * Agricultural use	Rhizosphere	0.197	0.01
	Roots	0.529	0.01
Plant dry weight	Rhizosphere	0.236	0.01
	Roots	0.45	0.01

Significance levels (*p*-value) are shown and asterisks (*p ≤ 0.05, **p ≤ 0.01, ***p ≤ 0.001) indicate a significant difference in factors between alpha diversity indexes and biotopes

**Fig. 3:**
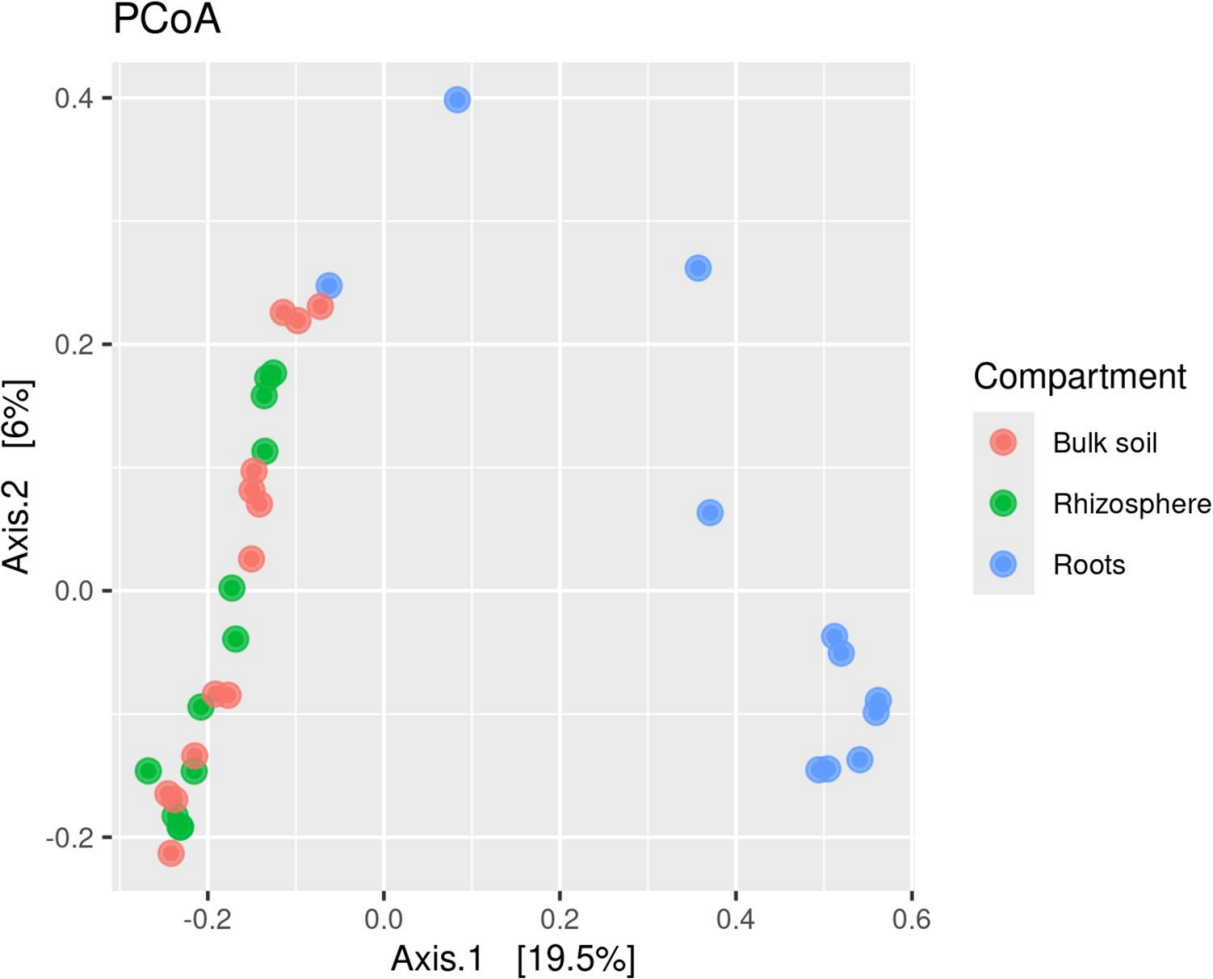
PCoA representation of the fungal communities in different biotopes. The rhizosphere, bulk soil, and root samples were subset to one per site

After subsetting the dataset to include only one root and one rhizosphere sample per site, no significant difference in beta-diversity was found between rhizosphere and bulk soil (*p* = 0.216). However, the root endosphere showed a significant difference in beta-diversity from both rhizosphere and bulk soil samples (*p* < 0.001 for both) (Fig. 3). Factors such as agricultural practices (*p* < 0.001) and the crop cultivated at the site (*p* < 0.001) significantly affected the beta-diversity of soil communities (Table 2). All soil parameters (total P, total N, total C, and total K) and precipitation volume were significant according to ANOVA performed on canonical correlation analysis results, with greater significance in the rhizosphere than in the roots. The plant phenotype significantly affected rhizosphere fungal communities but not the weight (Table 2). For each biotope, the significant factors with the highest *R*^2^ values were agricultural usage for the bulk soil and rhizosphere, and total P, C, N, and K for the roots (Table 2).

The Mantel test found spatial autocorrelation in soil communities (*p* = 0.02), but not in root communities (*p* = 0.097).

### Putative Dark Septates Endophytes

Putative dark septate endophytes, identified through BLAST analysis on the NCBI database using the term “dark septate,” comprised 20% of reads in roots and 8% in soils. When considering only ASVs likely belonging to dark septate endophyte species in the roots, the effect of precipitation level was not significant. Agricultural use and plant phenotype also did not show significance, but the crop cultivated in agricultural fields and all soil parameters except total C remained significant (Table S2). Root staining and microscopic observations clearly revealed that *M. sylvestris* roots are colonized by various fungal structures such as hyphae, microsclerotia, and haustoria-like structures without any apparent symptoms (Figure S1).

### Core and Hub Taxa

Since alpha-diversity and beta-diversity were not significantly different between bulk soil and rhizosphere samples, we treated the soil fungal communities as a single unified biotope for investigating core and hub taxa. One core ASV, attributed to the species *Fusarium equiseti*, was present in over 80% of both soil and root samples. Additionally, an ASV associated with *Alternaria subucurbitae* was detected in 80% of soil samples exclusively. This particular ASV was entirely absent from the roots and exhibited no correlation with any environmental factors. Furthermore, in more than 80% of root samples alone, an ASV related to the genus *Alternaria* was observed, with its abundance ranging from 0 to 30% of reads in each root sample. Notably, this ASV demonstrated a positive correlation with total K levels in the surrounding soil (*p* = 0.006).

A co-occurrence network analysis revealed a clear division of ASVs into two networks, one within the roots and the other within the soil (Figure S2). Within the soil network, ASV 62 exhibited the highest betweenness (11,210) and belonged to the species *Alternaria subcucurbitae*. All other taxa had a betweenness lower than 95% of 11,210, thus classifying ASV 62 as the sole hub taxa in the soil communities.

This ASV did not show any correlation with environmental factors (*p* > 0.05 for all factors). Similarly, in the roots, ASV 3620, belonging to the species *Fusarium equiseti*, emerged as the sole hub taxon, with a betweenness of 4238 and a significant negative correlation with precipitation levels (ASV 3620 in Table 3).

**Table 3:** List of ASVs correlated to precipitation and soil chemical factors in the rhizosphere and roots, along with their taxonomy. In the “origin” column, *Rh* denotes rhizosphere, and *Ro* denotes roots

ASV	Factor	Correlation	*p-*value	Origin	Family	Genus	Species
2	Precipitation	− 0.26	0.010	Rh	Nectriaceae	*Fusarium*	*F. equiseti*
15	Precipitation	− 0.22	0.031	Rh	Didymellaceae	*Stagonosporopsis*	
11	Precipitation	− 0.20	0.048	Rh	Lasiosphaeriaceae	*Immersiella*	
10	Precipitation	− 0.23	0.028	Rh	Nectriaceae	*Fusarium*	*F. equiseti*
3620	Precipitation	− 0.26	0.033	Ro	Nectriaceae	*Fusarium*	*F. equiseti*
3648	Precipitation	− 0.32	0.007	Ro	Olpidiaceae	*Olpidium*	
3640	Precipitation	− 0.33	0.006	Ro	Pleosporaceae	*Alternaria*	
3628	Precipitation	− 0.27	0.024	Ro	Nectriaceae	*Fusarium*	*F. equiseti*
16	Total C	− 0.23	0.024	Rh	Stachybotryaceae	*Stachybotrys*	*S. chartarum*
19	Total C	− 0.20	0.048	Rh	Cladosporiaceae	*Cladosporium*	*C. herbarum*
46	Total C	0.22	0.030	Rh	Pleosporaceae	*Alternaria*	
32	Total C	0.21	0.047	Rh	Pleosporaceae	*Alternaria*	*A. prunicola*
22	Total C	− 0.31	0.002	Rh	Cladosporiaceae	*Cladosporium*	*C. sphaerospermum*
14	Total C	− 0.20	0.049	Rh	Nectriaceae	*Fusarium*	*F. equiseti*
3635	Total C	− 0.32	0.008	Ro	Nectriaceae	*Dactylonectria*	*D. macrodidyma*
3678	Total C	− 0.30	0.014	Ro	Pleosporaceae	*Edenia*	*E. gomezpompae*
3	Total K	0.35	0.001	Rh	Ascobolaceae	*Ascobolus*	
5	Total K	0.30	0.003	Rh	Chaetomiaceae	*Botryotrichum*	
15	Total K	− 0.20	0.050	Rh	Didymellaceae	*Stagonosporopsis*	
12	Total K	0.31	0.002	Rh	Ascobolaceae	*Ascobolus*	
13	Total K	0.27	0.008	Rh	Ascobolaceae	*Ascobolus*	
28	Total K	0.49	0.000	Rh	Microascaceae	*Enterocarpus*	*E. grenotii*
3628	Total K	0.35	0.003	Ro	Nectriaceae	*Fusarium*	*F. equiseti*
2	Total N	− 0.21	0.041	Rh	Nectriaceae	*Fusarium*	*F. equiseti*
20	Total N	− 0.22	0.036	Rh	Cladosporiaceae	*Cladosporium*	*C. herbarum*
30	Total N	− 0.23	0.027	Rh	Nectriaceae	*Fusarium*	
22	Total N	− 0.26	0.012	Rh	Cladosporiaceae	*Cladosporium*	*C. sphaerospermum*
3617	Total N	− 0.29	0.015	Ro	Cladosporiaceae	*Cladosporium*	
2	Total P	− 0.38	0.000	Rh	Nectriaceae	*Fusarium*	*F. equiseti*
3	Total P	0.33	0.001	Rh	Ascobolaceae	*Ascobolus*	
5	Total P	0.26	0.012	Rh	Chaetomiaceae	*Botryotrichum*	
15	Total P	− 0.27	0.009	Rh	Didymellaceae	*Stagonosporopsis*	
16	Total P	0.49	0.000	Rh	Stachybotryaceae	*Stachybotrys*	*S. chartarum*
12	Total P	0.33	0.001	Rh	Ascobolaceae	*Ascobolus*	
25	Total P	0.27	0.009	Rh	Plectosphaerellaceae	*Gibellulopsis*	*G. nigrescens*
13	Total P	0.26	0.011	Rh	Ascobolaceae	*Ascobolus*	
28	Total P	0.20	0.049	Rh	Microascaceae	*Enterocarpus*	*E. grenotii*
10	Total P	− 0.35	0.000	Rh	Nectriaceae	*Fusarium*	*F. equiseti*
17	Total P	0.25	0.015	Rh	Lasiosphaeriaceae	*Cercophora*	*C. coronata*
30	Total P	0.45	0.000	Rh	Nectriaceae	*Fusarium*	
22	Total P	− 0.24	0.022	Rh	Cladosporiaceae	*Cladosporium*	*C. sphaerospermum*
14	Total P	− 0.32	0.002	Rh	Nectriaceae	*Fusarium*	*F. equiseti*

### Response and Association of Taxa to Environmental Factors

Eight ASVs were found to exhibit a significant negative correlation with precipitation levels, with four originating from the rhizosphere and four from the roots (Table 4). Among the four root ASVs, two were attributed to the genera *Alternaria* and *Olpidium*, while the other two belonged to the species *Fusarium equiseti*. Concerning soil chemical factors, eight ASVs showed correlations with total C, seven with total K, five with total N, and 15 with total P (Table 3). Of the 35 ASVs associated with soil chemical properties, only four were identified in the roots.

**Table 4:** List of 20 top predictors for discriminating between semi-arid and subhumid environment. Sites with more than 356 mm of rainfall by year were counted as subhumid, and sites with less than 356 mm as semi-arid

ASV	Origin	Gini	Predictor	Phylum	Genus	Species
302	Rhizosphere	0.76	Semi-arid	Ascomycota	*Lecythophora*	*L. canina*
79	Rhizosphere	0.68	Semi-arid	Ascomycota	*Preussia*	*P. polymorpha*
3737	Roots	0.67	Semi-arid	Ascomycota	*Edenia*	*E. gomezpompae*
384	Rhizosphere	0.6	Semi-arid	Ascomycota	Unknown	
4041	Roots	0.51	Semi-arid	Mortierellomycota	*Mortierella*	*M. exigua*
3785	Roots	0.46	Semi-arid	Basidiomycota	*Papiliotrema*	*P. fonsecae*
250	Rhizosphere	0.41	Semi-arid	Basidiomycota	*Conocybe*	*C. dunensis*
4156	Roots	0.39	Semi-arid	Mortierellomycota	*Mortierella*	
375	Rhizosphere	0.39	Semi-arid	Unknown	Unknown	
3717	Roots	0.37	Semi-arid	Ascomycota	*Gibellulopsis*	
3844	Roots	0.35	Semi-arid	Basidiomycota	*Malassezia*	*M. restricta*
3738	Roots	0.35	Semi-arid	Ascomycota	*Bisifusarium*	
84	Rhizosphere	0.35	Semi-arid	Ascomycota	*Ascobolus*	
3623	Roots	0.34	Subhumid	Ascomycota	*Alternaria*	
42	Rhizosphere	0.34	Semi-arid	Ascomycota	*Lasiobolidium*	*L. orbiculoides*
3893	Roots	0.34	Semi-arid	Mortierellomycota	Unknown	
1430	Roots	0.32	Semi-arid	Unknown	Unknown	
4789	Roots	0.32	Semi-arid	Ascomycota	Unknown	
200	Rhizosphere	0.32	Semi-arid	Ascomycota	*Dactylonectria*	*D. macrodidyma*
3734	Roots	0.31	Subhumid	Ascomycota	*Cladosporium*	

After a trimming process to retain ASVs representing more than 0.1% of the total sum of abundance, resulting in 923 remaining ASVs, a random forest model was utilized to identify optimal predictors for precipitation levels and agricultural practices. When constructing a model to distinguish root samples from semi-arid (annual precipitations < 356 mm) and sub-humid (> 356 mm) regions, 39 out of 176 samples (22%) were misclassified. Among these predictors, only one, present in the roots and belonging to *Cladosporium sp*., was more abundant in subhumid regions, while the rest were indicative of semi-arid regions. Notably, one of these semi-arid predictor ASVs belonged to the genus *Alternaria* and constituted a core taxon found in over 80% of roots (Table 4). Other predictors for the semi-arid climate in the roots belonged to the taxa *Edenia gomezpompae*, *Mortierella exigua*, *Mortierella* sp., *Malassezia restricta*, *Gibellulopsis* sp., and *Papiliotrema fonsecae*. In the rhizosphere, *Lecythophora canina*, *Preussia polymorpha*, *Conocybe dunensis*, *Ascobolus* sp., *Lasiobolidium orbiculoides*, and *Dactylonectria didyma* were the taxa represented by predictor ASVs. The list of the 20 most important taxa for predictions can be found in Table S3.

## Discussion

There was no significant difference in alpha-diversity between bulk soil and the rhizosphere, nor was there a difference in beta-diversity in semi-arid environments. Similarly, beta-diversity was not significantly different between bulk soil and the rhizosphere, suggesting that there is no selective recruitment of the fungal community in the rhizosphere of *M. sylvestris*. This observation aligns with findings in the bacteriome of *M. sylvestris* [15] and supports our initial hypothesis. Interestingly, in a study on sorghum under drought conditions, Gao et al. [23] found an increase in stochasticity in the rhizosphere mycobiome, which they linked to a decrease in selection by the host. Similarly, the rhizosheath mycobiome of herbaceous plants in the Namib Desert did not show a strong selection by the plant [24]. One possible explanation is that *M. sylvestris* may conserve resources that would otherwise be used to manage its rhizosphere environment. However, unlike the bacteriome of *M. sylvestris*, which showed higher alpha-diversity of bacterial communities in the rhizosphere, the rhizosphere in this study did not appear to serve as a refuge for fungal diversity, as alpha-diversities were not significantly different between the rhizosphere and bulk soil. This could be due to drought stress having a greater impact on bacterial communities than on fungal communities [25], and/or the lack of beneficial conditions for fungi in the rhizosphere.

Root and rhizosphere communities were highly sensitive to K, N, and C concentrations, while bulk soil communities remained unaffected. The bulk soil and rhizosphere communities responded to precipitation levels, unlike root communities, which showed no significant beta-diversity differences linked to precipitation. Additionally, rhizosphere communities were significantly influenced by the plant phenotype, whereas root communities were not. The sensitivity of rhizosphere communities to precipitation and plant phenotype, which is not observed in root communities, suggests that the roots of *M. sylvestris* provide refuge for fungal communities from drought stress. Alternatively, this could indicate that the selective filter imposed by the root biotope is stronger than the one imposed by dry conditions. Since some significant taxa within the roots, including those classified as “core taxa” and “hub taxa,” still respond to arid conditions, the second mechanism seems more likely. It is somewhat surprising that the rhizosphere communities differ between the two plant phenotypes, despite the lack of beta-diversity difference between bulk soil and rhizosphere. This may indicate selective recruitment of some taxa in the *M. sylvestris* rhizosphere under certain circumstances. This observation is supported by the fact that most of the taxa correlated with soil chemical parameters are found in the rhizosphere. However, this selective recruitment might be passive, resulting from modifications in the niche environment around the roots rather than active selective recruitment mediated by the plant [26, 27].

Despite the lack of precipitation effects on beta-diversity in the roots, the random forest model identified some ASVs that effectively predict aridity (25% error rate). This suggests a strong interaction between aridity levels and certain taxa within the roots, even if they are not the dominant taxa. Notably, one ASV from *Alternaria* sp., sometimes representing up to 30% of reads, was a predictor of drought and positively correlated with total K. Another *Alternaria* ASV also correlated with drought in the roots. Some members of the *Alternaria* genus, described as dark septate endophytes, have been shown to enhance plant resistance to salinity and drought [28]. It is plausible that *Alternaria* spp. play a role in *M. sylvestris*’s resistance to environmental stresses. Among the top 20 predictors associated with aridity, three ASVs belonged to the *Mortierellomycota* phylum, including one ASV in the *Mortierella* genus and another identified as *Mortierella exigua*. *Mortierella* species are known for their potential benefits to plants, such as enhancing P availability in the soil and exhibiting pathogen-suppressive activity [29]. Specifically, *M. exigua* has been noted as a potential agent for bioremediation of trace metals [30]. *Mortierella* species are also strongly associated with arid soils [31, 32]. While *Mortierella* spp. has not been directly linked to drought stress, our results suggest that species of this genus may have some potential in this context. The remaining ASVs associated with aridity are more related to pathogenicity, including those found in humans or animals. This suggests that these taxa may exhibit opportunistic pathogenic behavior, potentially stimulated by the drought stress experienced by plants in arid regions. Nevertheless*,* other members of the genus *Alternaria* have been described as pathogens in more studies than as dark septate endophytes [33]. Another genus correlated with drought in the roots, *Olpidium*, is also associated with pathogenicity and reduced drought resistance [34]. Thus, it is not possible to definitively affirm a positive or negative role for *Alternaria* spp. in *M. sylvestris* without further functional and experimental studies. Another ambiguous case is the species *Fusarium equiseti*, a core taxon present in both roots and soil samples, with two ASVs correlated with low precipitation. *F. equiseti* has been shown to benefit plant growth in high-salt soils [35], but it also causes root rot in chickpeas in Morocco [36]. Therefore, it is challenging to determine from this study alone which taxa are beneficial or pathogens. In general, the findings support hypothesis 2, suggesting that *M. sylvestris* plants host drought-responsive taxa in their roots.

Interestingly, another species of *Alternaria* genus, *A. subcucurbitae*, was identified as a core and hub taxon in the soil but was completely absent from the roots. These findings suggest that *Alternaria* and its associated microbial communities play a role in structuring the mycobiome of Moroccan arid environments. *Alternaria* is a cosmopolitan genus known for both saprophytic and pathogenic capabilities [36], including on Malvaceae plants [37]. It has been found to be the dominant genus in the foliar fungal endophytes of American plains environment, but with considerable intra-genus variation [38]. As previously mentioned, *Alternaria* spp. can also behave as a dark septate endophyte, and such endophytes have been shown to significantly influence the microbial communities of European forest soils [9]. *M. sylvestris* appears to be particularly associated with one specific species of *Alternaria*, which is not the most prevalent in the soil. However, it should be noted that the phylogeny of the *Alternaria* genus is still not well defined [36].

## Conclusion

*M. sylvestris* appears to host a few fungal taxa in its roots that may aid in coping with drought stress, with the most promising being a highly abundant ASV from the genus *Alternaria*. Other taxa of interest include *Fusarium equiseti* and taxa belonging to the *Mortierella* genus. In contrast, the rhizosphere mycobiome does not seem to be strongly selected by the plant, but it may respond to the physico-chemical characteristics of the soil. Further investigations, including the isolation of fungal endophytes from the roots of *M. sylvestris* and inoculation experiments, are needed to determine the effects of these endophytes on the growth and drought tolerance of the plant. Functional studies will also provide insights into the mechanisms employed by both partners to alleviate drought stress.

## Supplementary Information

Below is the link to the electronic supplementary material.

Supplementary file1 (DOCX 1748 KB)

## Data Availability

Raw reads are available in the NCBI repository under the BioProject PRJNA1122987.
