# Supplementary material for: Response of Alternaria and Fusarium Species to Low Precipitation in a Drought-Tolerant Plant in Morocco
Source: Microb Ecol. 2024 Oct 11;87(1):127. doi: 10.1007/s00248-024-02439-3 (PMC11469988; doi:10.1007/s00248-024-02439-3)
Supplement: Supplementary file 1 — Supplementary file1 (DOCX 1748 KB) [file 248_2024_2439_MOESM1_ESM.docx]

**Supplementary Information**

Table S1. Chemical and environmental features of the locations used for sampling.

| **Location** | **Crop** | **Latitude** | **Longitude** | **Precipitation** | **Total N** | **TotalP** | **Total K** | **Total C** |
| --- | --- | --- | --- | --- | --- | --- | --- | --- |
| Abou Jaad |  | 32.692 | -6.338 | 356 | 0.06 | 0.12 | 0.26 | 2.05 |
| Ait Ali | Pea | 32.496 | -6.2224 | 357 | 0.21 | 0.55 | 0.42 | 2.04 |
| Ait Ali | Beetroot | 32.496 | -6.2232 | 357 | 0.16 | 0.56 | 0.4 | 1.49 |
| Ait Ali | Faba Bean | 32.496 | -6.2227 | 357 | 0.14 | 0.48 | 0.37 | 1.79 |
| Ait Ali | Clover + Wheat | 32.496 | -6.2232 | 357 | 0.2 | 0.63 | 0.43 | 2.01 |
| Ait Hamou | Oignon | 33.488 | -6.611 | 528 | 0.17 | 0.07 | 0.57 | 1.66 |
| Ait Hamou | Oat | 33.488 | -6.611 | 528 | 0.18 | 0.06 | 0.64 | 2.08 |
| Benguerir |  | 32.15 | -7.907 | 291 | 0.18 | 0.09 | 0.48 | 1.7 |
| Berrechid |  | 33.285 | -7.612 | 343 | 0.16 | 0.31 | 0.4 | 2.14 |
| Bouskoura |  | 33.476 | -7.661 | 343 | 0.13 | 0.16 | 0.15 | 1.81 |
| Bouskoura | Barley | 33.476 | -6.836 | 343 | 0.18 | 0.09 | 0.16 | 1.77 |
| Firdaous |  | 33.476 | -6.836 | 528 | 0.06 | 0.05 | 0.05 | 0.69 |
| Nkhaila |  | 33.774 | -6.68 | 343 | 0.2 | 0.06 | 0.26 | 2.36 |
| Oued Laabid |  | 32.14 | -7.027 | 356 | 0.2 | 0.2 | 0.21 | 3.81 |
| Ouled Ayad |  | 32.212 | -6.8045 | 356 | 0.48 | 0.35 | 0.34 | 2.46 |
| Settat | Olive | 33.071 | -7.612 | 343 | 0.27 | 0.3 | 0.46 | 2.47 |
| Settat | Wheat | 33.071 | -7.612 | 343 | 0.31 | 0.57 | 0.92 | 2.96 |
| Tassaout |  | 32.148 | -7.262 | 356 | 0.23 | 0.07 | 0.26 | 2.2 |
| Zhiliga | Orange | 33.267 | -6.5621 | 420 | 0.25 | 0.1 | 0.16 | 2.08 |
| Zhiliga | Olive | 33.267 | -6.5621 | 420 | 0.25 | 0.1 | 0.16 | 2.08 |

Table S2 - *p*-values of the effects of various factors on the beta-diversity of putative dark septate in the roots.

| **Factor** | ***p*-value** |
| --- | --- |
| Total P | 0.003** |
| Total C | 0.07 |
| Total K | 0.017* |
| Total N | 0.018* |
| Precipitation | 0.341 |
| Agricultural use | 0,118 |
| Crop | 0.002** |
| Plant phenotype | 0.055 |
| Plant dry weight | 0.461 |

Table S3: List of the 20 most important taxa for predicting between agricultural and non-agricultural fields.

| **ASV** | **Origin** | **GINI** | **Predictor for** | **Phylum** | **Genus** | **Species** |
| --- | --- | --- | --- | --- | --- | --- |
| 2 | Rhizosphere | 1,2 | Non-agricultural | Ascomycota | Fusarium | equiseti |
| 3 | Rhizosphere | 0,8 | Agricultural | Ascomycota | Ascobolus | NA |
| 7 | Rhizosphere | 2,3 | Agricultural | Ascomycota | Cephaliophora | NA |
| 11 | Rhizosphere | 1,4 | Non-agricultural | Ascomycota | Immersiella | NA |
| 15 | Rhizosphere | 0,7 | Non-agricultural | Ascomycota | Stagonosporopsis | NA |
| 16 | Rhizosphere | 1,0 | Agricultural | Ascomycota | Stachybotrys | chartarum |
| 21 | Rhizosphere | 1,0 | Non-agricultural | Ascomycota | Alternaria | chlamydospora |
| 30 | Rhizosphere | 1,0 | Agricultural | Ascomycota | Fusarium | NA |
| 35 | Rhizosphere | 0,7 | Agricultural | Ascomycota | Preussia | flanaganii |
| 44 | Rhizosphere | 1,3 | Non-agricultural | Ascomycota | Plectosphaerella | niemeijerarum |
| 50 | Rhizosphere | 0,8 | Non-agricultural | Ascomycota | Stagonosporopsis | NA |
| 53 | Rhizosphere | 0,7 | Non-agricultural | Ascomycota | Stagonosporopsis | NA |
| 73 | Rhizosphere | 0,9 | Non-agricultural | Basidiomycota | Vishniacozyma | carnescens |
| 145 | Rhizosphere | 1,0 | Agricultural | Mucoromycota | Rhizopus | arrhizus |
| 3617 | Roots | 0,8 | Non-agricultural | Ascomycota | Cladosporium | NA |
| 3620 | Roots | 0,7 | Agricultural | Ascomycota | Fusarium | equiseti |
| 3623 | Roots | 1,0 | Agricultural | Ascomycota | Alternaria | NA |
| 3701 | Roots | 0,8 | Non-agricultural | Chytridiomycota | Unknown | NA |
| 3782 | Roots | 0,7 | Non-agricultural | Basidiomycota | Filobasidium | oeirense |
| 3844 | Roots | 1,2 | Non-agricultural | Basidiomycota | Malassezia | srestricta |

Fig. S1


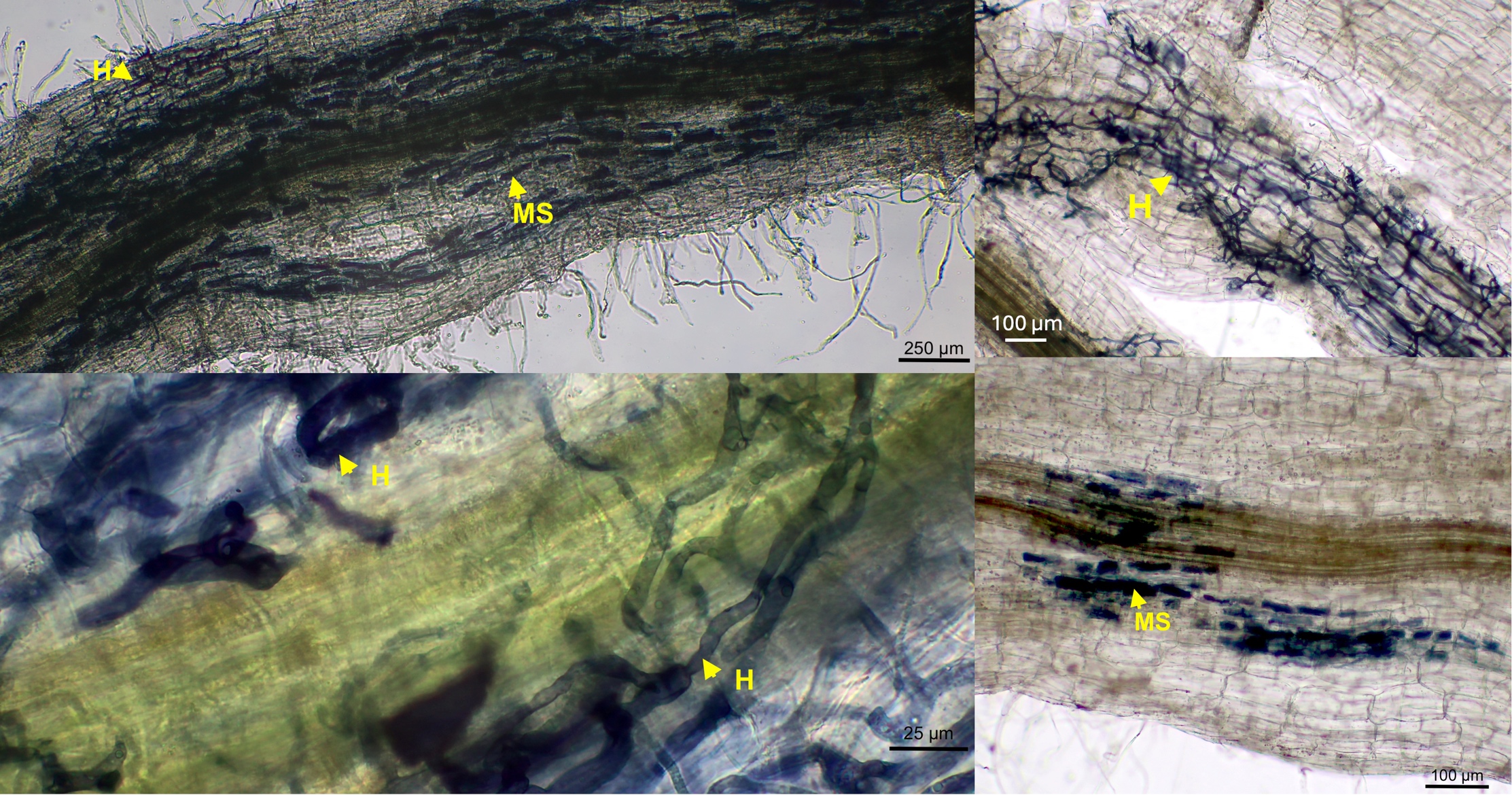


**Figure S1**. Microscopic images show fungal structures, including hyphae (H) and microsclerotia (MS), colonizing the roots of *M. sylvestris*.

Figure S2


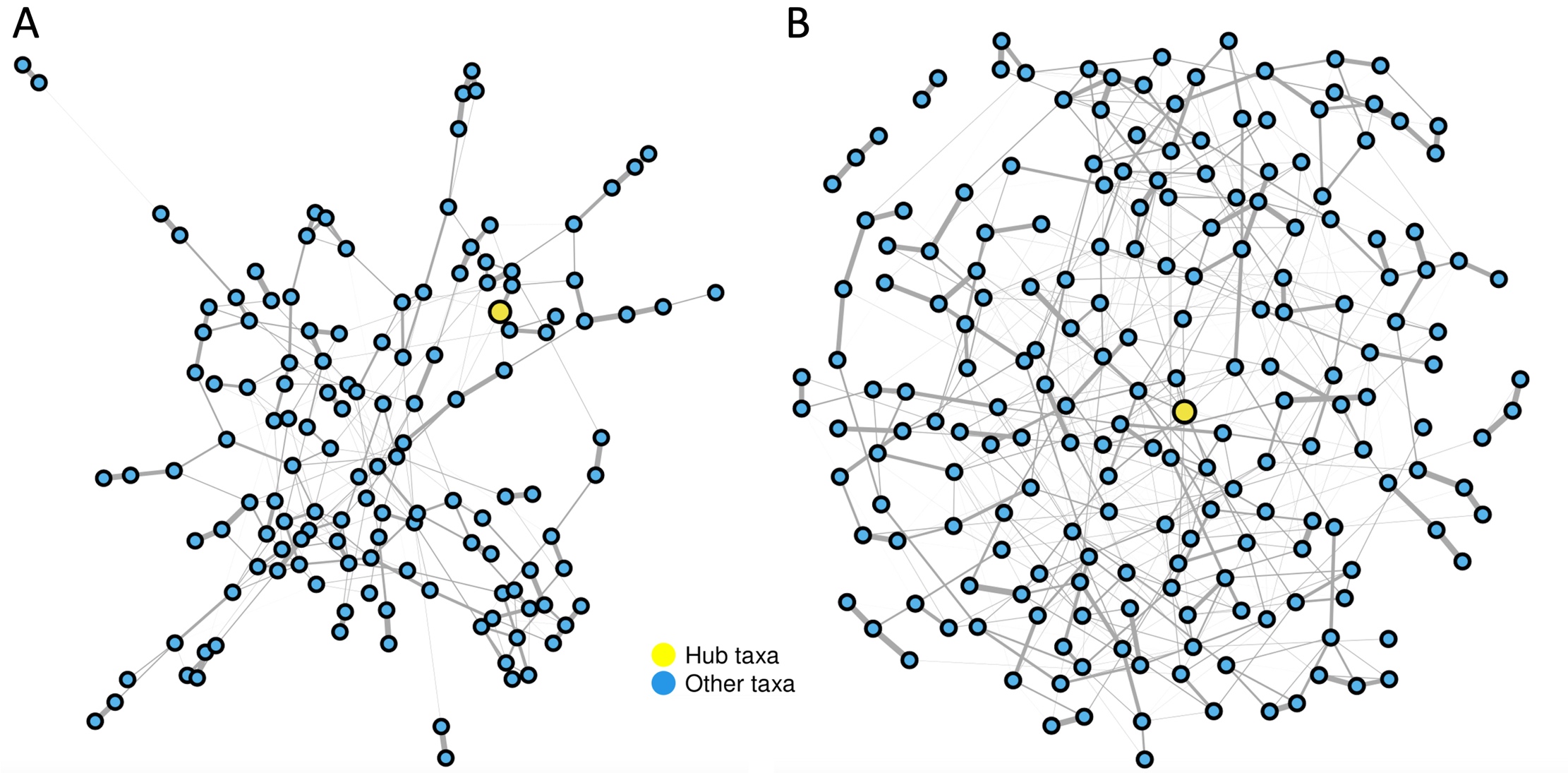


Figure S2. Co-occurrence networks of the most abundant ASVs in roots (A) and soil (B), with hub ASVs highlighted in yellow (*Fusarium equiseti* in roots and *Alternaria subcucurbitae* in soil).
